# Evolution in agricultural systems: Moving toward the understanding of complexity

**DOI:** 10.1111/eva.13490

**Published:** 2022-10-18

**Authors:** Leonor R. Rodrigues, Marta Montserrat, Sara Magalhães

**Affiliations:** ^1^ cE3c: Centre for Ecology, Evolution and Environmental Changes, Faculdade de Ciências Universidade de Lisboa Lisbon Portugal; ^2^ IHSM La Mayora‐UMA‐CSIC: Instituto de Hortofruticultura Subtropical y Mediterránea “La Mayora” Málaga Spain

**Keywords:** abiotic factors, agriculture, biological control, biotic interactions, genetic response, microbes, pesticide resistance

## Abstract

Agricultural fields are typically simplified ecosystems compared to natural sites, a characteristic that has long‐attracted researchers in Ecology and Evolution. In recent years, there has been a rising interest in understanding how agricultural systems are shaped by evolution in the context of changing agricultural practices by integrating biological information of crop systems. This editorial introduces the special issue “Evolution in agricultural systems,” incorporating the articles published within this issue into three general areas of research: phenotypic and genetic responses to the environment, biotic interactions and the role of microbes. Together, this body of work unveils unforeseen complexity at all levels, from microbes to trophic chains. Understanding such complexity is critical not only to better understand natural systems, but also if we wish to improve the sustainability of the food system.

*De Perto Ninguém é Normal – Caetano Veloso*

Agricultural systems are typically considered simplified ecological systems. First, agricultural fields are managed by humans that grow few species, especially in conventional agriculture, in which monocultures are common. Second, agriculture strongly relies on the use of pesticides, which have toxic effects not only on the targeted crop pests, but also on many other organisms embedded in the food webs within such agroecosystems (Geiger et al., 2010). This harmful effect of pesticides on biodiversity has motivated Rachel Carson to refer to the state of an agricultural landscape as a “Silent Spring” (Carson, 1962). Ironically, this simplicity has long attracted researchers in Ecology and Evolution for two main reasons: simple ecosystems are easier to track than highly complex systems; second, this simplicity allows testing key predictions in the field, rather than recreating ecological modules in the laboratory.

There are three iconic examples of this perspective. First, due to its simplicity, the genetic basis of pesticide resistance has long been viewed as a model trait to address the genetic basis of adaptation. Pesticides are designed by humans to kill crop pests and to specifically target particular molecules in such organisms, usually ion channels in the nervous system or neurotransmitters (Sparks & Nauen, 2015). This strong selection pressure and precise function is expected to select for an evolutionary response with a simple genetic basis (Orr & Coyne, 1992). Indeed, a wide range of studies has shown that the genetic basis of resistance to several insecticides in several crop pests is based upon a single allele (Roush & McKenzie, 1987; Weill et al., 2003), which is often the same even across different species (Ffrench‐Constant et al., 2004). Second, agricultural systems have been advocated as ideal to address the evolution of host range (Via, 1990), providing much needed empirical tests to a wide theoretical literature on the evolution of specialists and generalists (e.g., Kawecki, 1994; Levins, 1968; Ravigné et al., 2009; Van Tienderen, 1991). For instance, Sara Via, her collaborators, and several research groups thereafter, have provided a textbook example of the power of this approach, by unraveling the ecology, evolution, and genetic basis of specialization in the pea aphid (e.g., Hawthorne & Via, 2001; Via & Hawthorne, 2002). This research agenda is still ongoing and has been extended to other herbivorous arthropods (Sousa et al., 2019). Third, the fact that agricultural systems harbor few species facilitates the study of trophic interactions. Indeed, herbivore control generally relies on a single or few complementary species of natural enemies, often generating simple trophic chains, thus providing clear examples of trophic cascades in the wild (Polis et al., 2000). For example, the removal or addition of spiders in soybean monocultures had a clear impact on the degree of herbivory experienced by plants (Carter & Rypstra, 1995).

However, it is becoming increasingly clear that, if you look close enough, no system is simple. Indeed, the genetic basis of pesticide resistance is more complex than previously thought (Ffrench‐Constant, 2013; Labbé et al., 2007), adaptation to host plants do not follow simple rules even in simple systems (Laska et al., 2021; Magalhães et al., 2014), and trophic interactions are not always organized into a trophic chain, even in agroecosystems (Rosenheim, 1998; Rosenheim et al., 1995; Van Rijn et al., 2002). Moreover, agricultural landscapes are changing, with the general understanding that relying on pesticides and monocultures is not only environmentally detrimental, but also not optimal for pest control and plant yield in the long term (Janssen & van Rijn, 2021). Accordingly, several ecological and evolutionary studies are now being performed in complex agricultural landscapes (Marja et al., 2022; Scherber et al., 2010). This special issue – Evolution in Agricultural Systems – reflects this acknowledgement. Indeed, and even though this was not an explicit criterium, all the contributions we have received explore complexity at some level, from the genetic basis of phenotypes to ecological interactions, including previously overlooked players such as microbes.

1. Complexity in the phenotypic and genetic response to the environment
  - Resistance to xenobiotics

As mentioned earlier, the notion that resistance to xenobiotics has a simple genetic basis has been challenged. Even in systems where resistance is known to be encoded in specific regions of the genome, different mutations may confer such resistance. For example, Einspanier et al. (2022) identified 5 different mutations conferring resistance to Succinate Dehydrogenase Inhibitors and Demethylation Inhibitors within their target site (Cyp51) in 43 isolates of the *Alternaria solani* fungus sampled in five European countries. These isolates were grouped into 7 genotypes, but there were no signs of population structure according to geographical location. Moreover, they found evidence of recombination in this fungus, typically considered a clonal pathogen. Still, despite these lines of evidence for considerable gene flow among populations, there was no link between specific mutations and genotypes or recombination events, leading to the conclusion that the same mutations probably arose multiple times independently. This result suggests that the existing mutations were under strong selection, which is expected to select for a simple genetic basis for pesticide resistance (Orr & Coyne, 1992; Roush & McKenzie, 1987). Importantly, although here a single mutation is sufficient to entail pesticide resistance in each population, the fact that different mutations confer resistance in different populations may lead to complex dynamics in resistance and its genetic underpinnings (Labbé et al., 2007).

An even more complex scenario arises from a study on pesticide resistance in the Colorado Potato Beatle, *Leptinotarsa decemlineata* (Z. P Cohen, Y. H. Chen, R. Groves, S. D. Schoville, unpublished data). Resistance to pesticides has been studied for many decades in this major crop pest (Alyokhin et al., 2008), providing examples of a simple genetic basis for resistance to different pesticides (Kim et al., 2007). Yet, using whole genome sequencing, Z. P. Cohen, Y. H. Chen, R. Groves, S. D. Schoville (unpublished data) reports a highly polygenic basis for resistance to neonicotinoids. Moreover, a comparison between resistant populations from Wisconsin and Long Island revealed that the single nucleotype polymorphism (SNPs) associated to resistance are mostly population specific. Although a functional validation of such SNPs may lead to the exclusion of some, this data strongly supports a complex evolutionary history of pesticide resistance in these beetles.

A possible explanation for this heterogeneity in the response to xenobiotics may lie in the variability stemming from the host plants that herbivores colonize. Indeed, different host plants exert different selection pressures upon herbivores, often leading to the formation of host races (Drès & Mallet, 2002; Magalhães et al., 2007), and pesticide resistance is associated with some plants rather than others (Dermauw et al., 2013; Singh et al., 2021). Roy et al. (2022) build on this previous work and show that the spatial and temporal dynamics of four target‐site mutations conferring resistance to carbamates, pyrethroids, and neonicotinoids in the aphid *Myzus persicae* are associated to different host plants and reproductive modes. Possibly, the selection for such mutations conferring resistance is host‐specific and restricted gene flow between host plants and reproductive modes have created the conditions for such different mutations to arise.

b Plant responses to environmental variables

Most crops have been modified by artificial selection along centuries, to increase their yield and expedite their maintenance. Scientists have been fascinated by the genetic basis of the phenotypes that make certain plants great crops. For example, the seminal work of George Beadle, and then John Doebleys' group, has unequivocally shown that few genetic changes separate maize from teosinte, its wild ancestor (Doebley, 2004). Research on this topic, besides addressing the genetic basis of domestication, also tackles the genetic basis of plant responses to environmental variables (Flood & Hancock, 2017), particularly that of cereals, for which several strains and panels of recombinant inbred lines are available (e.g., Lasky et al., 2015; Russell et al., 2016). Most of these endeavors concern plant responses to environmental variables associated with climate change, such as temperature or drought (Gupta et al., 2020; Raza et al., 2019). Again, here, complexity is the rule rather than the exception.

Ćalić et al. (2022) measured the selection gradients associated to drought resistance in rice (*Oryza sativa*), by exposing seeds from more than 200 accessions of Indica or Japonica, the two rice geographical races, to wet or dry conditions in the field. They found a strong selection for early flowering in both varieties and both environments. Also, several traits showed significant selection gradients in the wet environment, but not under dry conditions. Using a genome wide association study (GWAS) approach, they measured genetic variances and covariances among traits (i.e., G‐matrices) based on SNPs. They found significant (broad‐sense) heritability and positive genetic correlations among traits, indicating a complex genetic basis for the response to these environments. The strong selection pressure associated to these traits, together with these positive correlations, begs the question of what maintains genetic diversity in these traits.

Similarly, shade avoidance in wheat (*Triticum turgidum*) was shown to have a complex genetic basis (Colombo et al., 2022). Using a panel of 180 recombinant inbred lines, this study revealed that the response to shade is consistently associated with 6 QTLs underlying plant height. Moreover, in three of them, genotypes associated with short plants systematically expressed reduced shade avoidance, suggesting a positive genetic correlation between plant height and plant height plasticity. This study contributes to the ongoing debate on the relationship between the genetic basis of traits and their plasticity (Lafuente & Beldade, 2019).

c Traits that make a good biocontrol agent are not what they used to be

The previous two examples show that, in agricultural systems as in other environments, the complexity is not only in the genetic basis of adaptation, but also in the phenotypic response, as many traits are simultaneously modified by a single environmental variable. Such complexity may be used by researchers as a tool to improve the performance of biocontrol agents (Leung et al., 2020; Montserrat et al., 2021). For instance, most studies aiming at improving the performance of biocontrol agents via artificial selection concern only life‐history traits (Lirakis & Magalhães, 2019), despite having long been recognized that behavioral traits strongly contribute to their performance (Luck, 1990). Lartigue et al. (2022) present a novel approach to improve top‐down control of agricultural pests based on exploiting the genetic variability of personality traits in the parasitoid *Trichogramma evanescens*, a known biological control agent. Using 24 near‐isogenic lines, they found significant (broad‐sense) heritability for boldness, activity, and exploration, traits that are likely associated with a high efficiency of searching for prey (Bielza et al., 2020; Rodrigues et al., 2016). Although, in this case, exploration is traded off with fecundity, this approach opens a new window of opportunities to the field of biological pest control as it introduces the possibility for the artificial selection of behavioral traits.

2 Complex interactions

The shade avoidance analyzed in Colombo et al. (2022) can be viewed as a trait in response to an abiotic selection pressure – the absence of light –but also to a biotic factor – competition for access to light. In fact, much of the evolutionary responses observed in both natural and agricultural systems are triggered by interactions with individuals, be it con‐ or heterospecifics. These “others” represent most of the complexity found in such systems. In fact, even in a field with monocultures that is supposedly not attacked by herbivores, conspecific plants exert complex selection pressures on each other. Intraspecific interactions can range from competition to cooperation depending not only on external conditions but also on the genotypic composition of the population where such interactions occur (Gardner et al., 2011; Hamilton, 1963; Kéfi et al., 2008).

In crop fields, initial trait values, plant density, and relatedness are key factors in defining which type of interaction will prevail, but the outcome of their interplay remains elusive (Montazeaud et al., 2020). Biernaskie (2022) provides an overview of the theory underlying kin selection and how it might affect overall plant yield. He suggests three breeding designs that make use of kin selection to increase plant fitness (hence yield): artificial selection for individuals with less competitive phenotypes, selecting groups of individuals with higher fitness than other groups, and identifying and selecting cooperative traits.

Most crop fields, however, are composed of more than one plant species at some spatial and temporal scale. This heterogeneity has implications for plant–plant interactions, but also for the interaction between herbivores and plants. Indeed, exposure to several plant species is expected to select for generalist herbivores, unless a strong trade‐off prevents adaptation to more than one plant species (Kassen, 2002; Levins, 1968). This, in turn, may have further consequences for herbivore performance on yet other plants, having thus a strong impact on their host range (Gould, 1979). The wheat curl mite (*Aceria tosichella*), a species long considered a generalist, is actually a complex of cryptic species with variable host ranges (Skoracka et al., 2013). Using a generalist lineage thereof, Skoracka et al., 2022 tested whether evolving on one or two plant species affected the mites' host range. After 45 and 60 generations of experimental evolution on either wheat, barley, or on an alternation between the two, mites were exposed to each of these plants and to brome and rye. The performance of mites evolving on a single plant species was higher on that plant than that of mites evolving on alternating plant species, while the latter performed better on novel host plants. This suggests that having more than one plant species in a field may slow down the adaptation of pests to crops, but it increases the risk that they colonize several plants.

Unveiling complexity has also important consequences for the third trophic level of agricultural systems, composed of natural enemies of herbivores. Indeed, the concept of biological control is anchored on that of trophic cascades, which posits that the third trophic level alleviates pressure on the first by consuming the second (the so‐called “green world hypothesis,” Hairston et al., 1960). However, such cascades generally work in simplified systems, which may not be the case of most agricultural systems. In fact, several natural enemies of crop pests may co‐occur in agricultural fields, and their ability to coexist and act additively or synergistically against herbivores may depend on abiotic conditions (Guzmán et al., 2016), the occurrence of intraguild predation (Snyder & Ives, 2001) and/or the presence of alternative prey (Cardinale et al., 2003). How evolution may affect the outcome of such complex interactions is difficult to predict. Sentis et al. (2022) discuss several features of evolutionary theory that can shed light on this issue.

3 The role of microbes

Agricultural fields clearly harbor more species than the minimal three that compose a trophic chain. However, such trophic complexity is just the tip of the iceberg. Indeed, soils, plants, herbivores, and their predators all contain microbes that may act as hidden players in the system and thus affect its evolution. Although the study of microbiomes is currently buoyant, their role in the ecological and evolutionary outcomes in agricultural systems remains mostly elusive.

By and large, the impact of the soil microbiome on agriculture is more studied than that of other components of agricultural systems. For example, nitrogen fixation by bacteria was discovered as early as 1901 by Martinus Beijerinck. This process is key to agriculture productivity (and basically to all life on earth), as it converts the inert triple‐bonded N2 into ammonia (NH3), making nitrogen available to the biosynthesis of amino acids and nucleic acids. The synthetic version of this conversion, known as the Haber–Bosch process, curiously also discovered in the early 20^th^ century, is at the basis of synthetic fertilizers (and of explosives too…), which allowed a major boost in agricultural productivity. Still, despite this early recognition of the importance of bacteria to plant biosynthesis, and thus agriculture, the myriad of possible roles of micro‐organisms in agricultural systems is only starting to unfold.

Recently, there has been a shift in the study of the interaction between plants or animals and bacteria, from one–on–one interactions to considering the microbiome as a whole (Gerardo et al., 2020; Hawkes et al., 2020). Yet, whereas addressing the role of a single bacteria species may be simplistic, studying that of whole microbiomes may be intractable. Hence, we need to understand how small groups of micro‐organisms interact among them and how together they interact with plants so that we can extract rules that widen our knowledge of how such interactions affect evolution in agricultural systems.

Two manuscripts in this special issue provide novel insight on the role of plant‐microbe interactions in agriculture. Klein et al. (2022) discuss how addressing such interactions from the microbe perspective may illuminate mechanisms fostering the beneficial effects of microbes on plants. Such mechanisms pertain both to the abiotic conditions in which plant‐microbe interactions take place and to the interactions among microbes themselves. Denison and Muller (2022) propose novel methods to measure the costs and benefits of harboring particular combinations of microbial strains for plant productivity.

Microbes are also important for the interaction between plants and herbivores (Frago et al., 2012), and they may even play an important role in determining whether organisms are considered pests (Hosokawa et al., 2007). While knowledge on the role of microbes in the outcome of species interactions is well characterized in some systems, the factors accounting for the composition and structure of this microbiome are mostly unknown. Ravigné et al. (2022) addressed this issue using bacterial gut microbiota of 8 sympatric Tephritidae flies from the Reunion Island, teasing apart the contributions of host phylogeny, specialization, and sampling environment (lab vs. field). The strong effect of host phylogeny and the relatively weak impact of the host feeding strategy and sampling environment on microbiome composition, suggests that the gut microbiome is vertically transmitted or strongly filtered from the environment in these species.

Likewise, the interaction between herbivores and their natural enemies may be modulated by the presence of microbes (Frago et al., 2017; Su et al., 2013). For example, aphids that harbor symbionts can become less conspicuous to parasitoids because such symbionts affect the emission of plant volatiles that attract parasitic wasps (Frago et al., 2017). Still, not all variation in the success rate of aphid biocontrol by parasitoids is explained by aphid populations differing in their heritable elements (endosymbiont‐ or endogenous‐based), as shown in Beekman et al. (2022). In turn, parasitoids used as biocontrol agents of crop pests also harbor symbionts that affect several traits in their hosts (Dicke et al., 2020). The wealth of existing studies clearly shows that the endosymbiotic community of both crop pests and their natural enemies may affect the outcome of biological control, a possibility that is analyzed in Sentis et al. (2022).

4 Are we harnessing complexity or is complexity harnessing us?

The studies we have gathered show that the more complexity is unveiled, the more we realize the orders of magnitude of such complexity. Still, we are certainly moving forward in our understanding of agricultural systems. First, we are realizing that the complexification of trophic links does not necessarily imply more complex interactions. For example, predators often tend to avoid engaging in predator–prey interactions when they co‐occur (Roubinet et al., 2015; Torres‐Campos et al., 2020). Second, decades of curiosity‐driven studies in agricultural fields have been contributing not only to finetune predictions, but also to develop diagnostic methods and tools to better manage agricultural fields (Mavridis et al., 2022). For example, Fritz (2022) discusses the possibility of using whole genome scanning, by means of genomic approaches and bioinformatic tools, to monitor, in real time, the dynamics of resistant genotypes in agroecosystems.

Importantly, it is becoming increasingly clear that the conventional mode of producing food is unsustainable from an environmental, social, and economic perspective. Indeed, evidence is accumulating that reducing the use of pesticides, increasing crop diversity and that of natural enemies leads to more effective pest control, better ecosystem services, and higher yields (Dainese et al., 2019; Janssen & van Rijn, 2021; Snyder, 2019; Windsor et al., 2021). Alternative production methods, such as organic farming, agroecology, agroforestry, etc., all imply that agricultural fields will become increasingly complex. Understanding such complexity is thus vital, not only to satisfy our curiosity and to serve as a stepping stone to understand more natural systems, but also to ensure the sustainability of the food system, which is at the core of the 2030 United Nations Agenda for sustainable development (Nations, 2015).

## CONFLICT OF INTEREST

The authors declare no conflict of interest.

## Data Availability

None.
